# High-Throughput Absolute Quantification Sequencing Reveals that a Combination of Leguminous Shrubs Is Effective in Driving Soil Bacterial Diversity During the Process of Desertification Reversal

**DOI:** 10.1007/s00248-022-02151-0

**Published:** 2022-12-10

**Authors:** Wangsuo Liu, Kaiyang Qiu, Yingzhong Xie, Yeyun Huang, Ruixia Wang, Haichao Li, Wenfen Meng, Yi He, Yayuan Li, Haiquan Li, Pengbo Zhao, Yi Yang

**Affiliations:** 1 School of Agriculture, Ningxia University, Yinchuan, 750021 Ningxia China; 2 Ningxia Grape Wine and Desertification Prevention Technical College, Yinchuan, 750199 Ningxia China; 3 Ningxia Grassland and Animal Husbandry Engineering Technology Research Center, Yinchuan, 750021 Ningxia China; 4 Breeding Base for State Key Laboratory of Land Degradation and Ecological Restoration of Northwest China, Yinchuan, 750021 Ningxia China; 5 Ningxia Administration of Baijitan National Nature Reserve, Lingwu, 750400 Ningxia China

**Keywords:** Combination of leguminous shrubs, Soil bacterial diversity, Absolute quantification sequencing, Edaphic factor, Sand-fixing afforestation, Straw checkerboard

## Abstract

Desertification leads to the extreme fragility of ecosystems and seriously threatens ecosystem functioning in desert areas. The planting of xerophytes, especially leguminous shrubs, is an effective and common means to reverse desertification. Soil microorganisms play a crucial role in nutrient cycling and energy flow in ecosystems. However, the effects of introducing leguminous shrubs on soil microbial diversity and the relevant mechanisms are not clear. Here, we employed the high-throughput absolute quantification 16S rRNA sequencing method to analyze the diversity of soil bacteria in sand-fixing areas of mixed shrublands with three combinations of shrubs, i.e., *C. korshinskii* × *Corethrodendron scoparium* (CaKCoS), *C. korshinskii* × *Calligonum mongolicum* (CaKCaM), and *C. scoparium* × *C. mongolicum* (CoSCaM), in the south of the Mu Us Sandy Land, China. This area suffered from moving dunes 20 years ago, but after introducing these shrubs to fix the dunes, the ecosystem was restored. Additionally, the effects of soil physicochemical properties on soil bacterial composition and diversity were analyzed with redundancy analysis (RDA) and structural equation modeling (SEM). It was found that the Shannon index of soil bacteria in CaKCoS was significantly higher than that in CaKCaM and CoSCaM, and the abundance of the dominant phyla, including Actinobacteria, Proteobacteria, Acidobacteria, Chloroflexi, Planctomycetes, Thaumarchaeota, Armatimonadetes, candidate_division_WPS-1, and Nitrospirae, increased significantly in CaKCoS and CaKCaM compared to that in CoSCaM. RDA showed that the majority of soil properties, such as total nitrogen (TN), available potassium (AK), N:P ratio, soil moisture (SM), and available phosphorus (AP), were important soil environmental factors affecting the abundance of the dominant phyla, and RDA1 and RDA2 accounted for 56.66% and 2.35% of the total variation, respectively. SEM showed that the soil bacterial α-diversity was positively affected by the soil organic carbon (SOC), N:P ratio, and total phosphorus (TP). Moreover, CaKCoS had higher SM, total carbon (TC), total potassium (TK), and AP than CaKCaM and CoSCaM. Collectively, these results highlight a conceptual framework in which the combination of leguminous shrubs can effectively drive soil bacterial diversity by improving soil physicochemical properties and maintaining ecosystem functioning during desertification reversal.

**Graphical Abstract:**

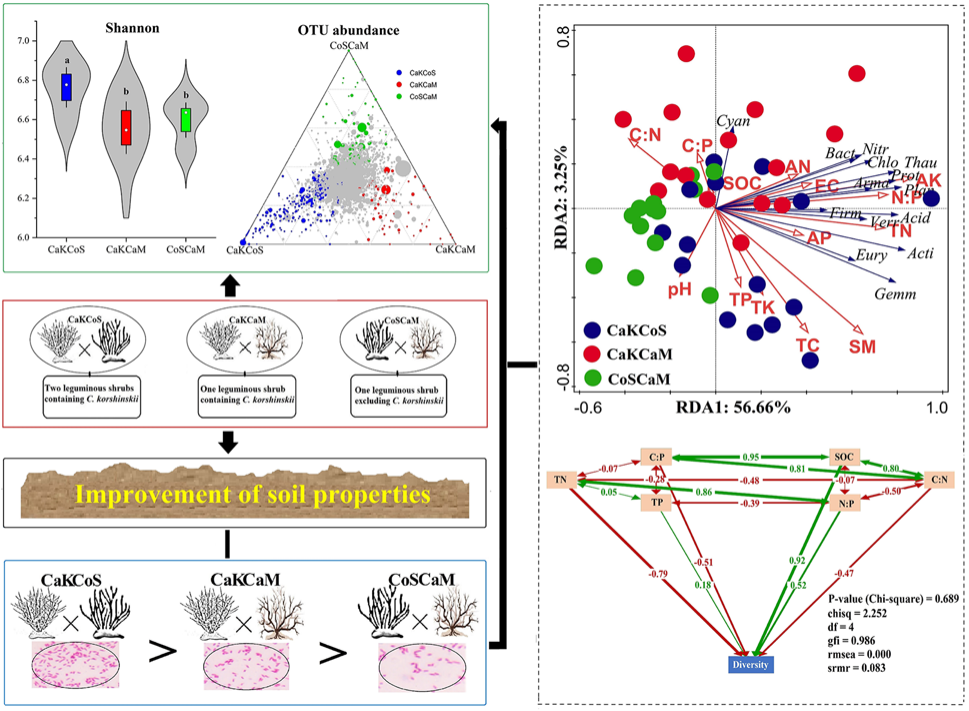

**Supplementary Information:**

The online version contains supplementary material available at 10.1007/s00248-022-02151-0.

## Introduction

Desertification is a worldwide environmental problem and poses a great threat to ecosystems in dryland regions [91]. The increasing expansion of desertification not only limits economic development and reduces people’s quality of life but also causes vegetation degradation and ecological imbalance [10]. Desertification results in serious soil erosion, low vegetation coverage and sparse ground surface soil, and a continuous reduction in soil nutrients and moisture, resulting in barren sandy land and frequent sandstorms [46, 91]. These changes have strongly affected the ecological processes and functions of the sandy land ecosystem [77, 105, 111]. Thus, desertification control has captured great attention from all over the world. Different means of reversing desertification have been used in different regions of the world, for example, in the Mediterranean region, the establishment of shrubs is a crucial step in the process of desertification reversal [7, 47]. In Central Asian deserts, *Haloxylon* is widely used in contributing to desertification reversal due to its excellent sand-fixing properties [54]. In the Chihuahua deserts of South America, shrubs are often identified as pioneer “nurse” plants, forming species-rich patches [70]. In the sandy soil of the northern Sahara in Algeria, the richness of plant species increases with increasing vegetation coverage, and the protective effect of vegetation plays an important role in the restoration of sandy soil [48]. Following the conference on desertification control sponsored by the United Nations in 1977, the Chinese government has taken positive actions to prevent and control desertification, including ecological restoration projects such as the Three-North Shelter Forest Program, the “Grain for Green” Program, and the Natural Forest Protection Program, aimed at saving these fragile desertified areas [45]. For example, in northern China, the legume *C. korshinskii* was used to control large areas of sandy land through sand fixation restoration [83, 85]. After several stages of afforestation restoration practices, desertification in northern China has been effectively controlled and has achieved remarkable feedback [46].

Many studies have shown that afforestation projects, such as the introduction of drought-tolerant plants and the sand-fixing method, have effectively stopped the process of desertification and have gradually showed a trend of desertification reversal [47, 59, 100, 111, 114, 115]. Among them, straw checkerboard barrier measures have been widely applied to control the spread of moving dunes, allowing for sand fixation all over the world [58, 82]. One of the effective applications is the planting of xerophytic shrubs in straw checkerboards [36, 74, 104]. These sand-fixing patterns of combining xerophytic shrubs with straw checkerboards can effectively promote the physical and chemical properties of the soil [36, 74], and the nursing effect of the shrub canopy can enhance the development of herbaceous plant species [39], thus forming fascinating and resource-rich harbors known as “islands of fertility” [1, 6]. In extremely arid and semiarid areas, shrubs, especially nitrogen-fixing leguminous shrubs [40, 65], promote the formation of “islands of fertility” and improve soil nutrient accumulation, which is an extensive method of land degradation restoration [66, 87]. The leguminous shrub *Retama sphaerocarpa* is a commonly used species [55]. Nitrogen (N) fixation by leguminous shrubs, such as *Caragana korshinskii* [93, 95, 103], has been shown to improve soil N content and accumulate rich carbon (C) sources from leaf litter and root exudates. Plants of *Caragana* and *Corethrodendron* are xerophytic leguminous shrubs and are the dominant plants with the largest afforestation area in northern China; they are the most common shrubs used in sand fixation [5, 85]. Long-term afforestation practices and research have indicated that the leguminous shrub *C. korshinskii* has a strong ability for regrowth [17] and water acquisition [88], as well as excellent performance regarding sand fixation [18, 19]. Therefore, the sand-fixation mode of planting leguminous shrubs within straw checkerboards is of great significance for understanding the underlying mechanism of soil microecology in sandy areas with harsh environments.

Soils harbor diverse microbial communities, among which bacteria and fungi play an essential role in regulating C and N cycling processes for ecosystem functioning [28]. The diverse soil microbiota is frequently accompanied by different vegetation types in sandy land because aboveground vegetation can not only effectively improve soil texture, physicochemical properties, and the microenvironment but also input C stores from litter and root exudates [76, 95, 103]. Bacteria are an important part of the ecosystem that participate in nutrient cycling, fight against pathogenic microorganisms, improve the health of plants, and enhance ecological functions [22, 79]. In desert ecosystems, planting xerophytic plants can effectively enrich the soil bacterial community and diversity. Cao et al. [5] suggested that xerophytic plants, including *C. microphylla*, *Artemisia halodendron*, *Hedysarum fruticosum*, *Pinus sylvestris* var. *mongolica*, *Populus simonii*, and *Salix gordejevii*, have been artificially planted for sand fixation for 32 years, and the soil bacterial community composition was similar to that of the native community [110]. Zhou et al. [116] found that the rhizocompartments of two leguminous shrubs (*Hedysarum scoparium* and *H. mongolicum*) strongly influenced the soil bacterial communities and had filtration and enrichment effects on beneficial soil microbiota. Mediterranean wild leguminous shrubs, with their N-fixing rhizobia and mycorrhizal properties, play the most important role in contributing to land restoration and desertification reversal [7]. Most of the shrubs involved in sand-fixing afforestation are mainly legumes that can fix atmospheric N and may be the key factor affecting the formation of beneficial soil bacteria in the process of desertification reversal [60]. Evidence has been found that afforestation with the legumes *C. korshinskii* and *Robinia pseudoacacia* effectively increased the soil bacterial abundance and diversity compared with abandoned land on the Loess Plateau of China, and the Proteobacteria phylum gradually evolved into the dominant phylum [63], mainly due to the change in the soil N:P ratio [61, 64, 107, 108]. Therefore, the formation of soil nutrient pools may be mediated by legumes to a large extent and exert a large influence on the evolution of soil bacterial communities.

However, our knowledge about the effects of the combination of leguminous and nonleguminous shrubs on soil bacterial diversity during desertification reversal is still poor. Given the contribution of leguminous shrubs to soil properties and microhabitats in sandy land, as well as the excellent performance of the xeric leguminous shrubs in afforestation in sand-fixing areas, we hypothesized that the combination of leguminous shrubs in sand-fixing afforestation is more effective in ameliorating soil physical and chemical properties and enriching soil bacterial diversity than the combination of one leguminous and nonleguminous shrub after the long-term restoration of sandy land. The objective of this study is to test the following hypothesis: the combination of two leguminous shrubs effectively improves soil bacterial diversity by ameliorating soil environmental factors such as soil physical properties and nutrient contents during the process of desertification reversal. Our study contributes to addressing the issues of integrating strategies of shrub afforestation in nutrient-deficient sandy lands all over the world.

## Materials and Methods

### Study Area and Sampling Design

**Fig. 1:**
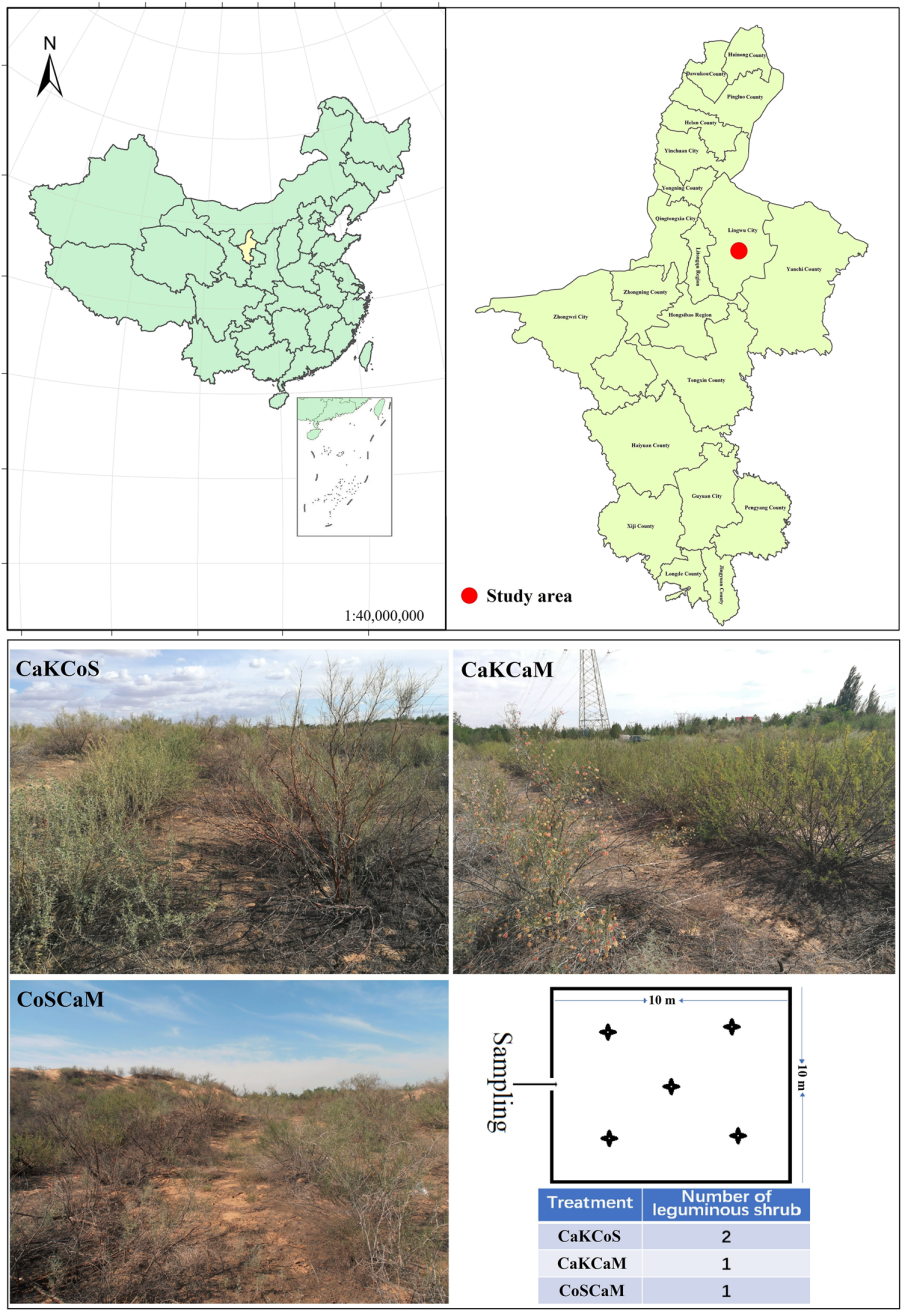
Location and photographs of the study area and sampling design of the three combinations of leguminous shrubs

The study area was located in the Baijitan National Nature Reserve in Ningxia and belongs to the core area for sand fixation via straw checkerboards combined with shrubs in the south of the Mu Us Sandy Land, China (37°58′26″N, 106°24′03″E) (Fig. 1). The Mu Us Sandy Land is one of the largest sandy lands in China and is characterized by a fragile ecological environment and drought conditions due to desertification [91]. This area has a typical continental monsoon climate, and the annual average precipitation is approximately 230 mm and is mostly concentrated from July to September. The average annual accumulated temperature is approximately 3334.8 °C, with an average annual temperature of 6.7–8.8 ℃ and a frost-free period of 157 days. The soil type in this area is mainly aeolian sandy soil, and the vegetation is sparse. In 2001, moving dunes were covered with straw checkerboards (half-buried in sand) with each checkerboard covering an area of 1.0 m × 1.0 m. In the checkerboard, xeric shrubs dominated by *C. korshinskii*, *C. scoparium*, *C. mongolicum*, and *C. fruticosum* var. *mongolicum* were planted in 2002 with a row spacing of 2 m × 2 m. After years of combating desertification, the sand-fixing model of straw checkerboard-coupled vegetation was successfully implemented, and the moving dune was effectively fixed. Currently, it is considered a typical sand-fixing area worldwide. For example, the sand-fixing model of planting two xerophytic shrubs on a checkerboard is widespread in northern China [20]; in particular, leguminous shrubs are widely used in sand fixation due to its N fixation capacity [23], and the most typical representative shrubs are *C. korshinskii* [17], *C. scoparium* [5], and *Corethrodendron mongolicum* [116].

It was observed that these sand-fixing measures effectively prevented the shift of dunes and the occurrence of sandstorms [102, 114]. Thus, we selected 3 typical combinations of shrubs for sand-fixing afforestation conducted in 2002 as our experimental treatments on the southwestern margin of the Mu Us Sandy Land of China, that is, *C. korshinskii* and *Corethrodendron scoparium* mixed shrubland (2 m × 2 m, row pitch), defined as two leguminous shrubs containing *C. korshinskii* (CaKCoS),*C. korshinskii* and *Calligonum mongolicum* mixed shrubland (2 m × 2 m, row pitch), defined as one leguminous shrub containing *C. korshinskii* (CaKCaM); and *C. scoparium* and *C. mongolicum* mixed shrubland (2 m × 2 m, row pitch), defined as one leguminous shrub excluding *C. korshinskii* (CoSCaM). Three plots were established for each combination of shrubs (treatment), and approximately 1.2 hm^2^ of each plot was selected as the observation area, with a distance of 1 km from each plot. Each plot included 5 sampling quadrats of 10 m × 10 m. None of the areas had been subjected to anthropogenic disturbances since their establishment (Fig. 1).

### Soil Sampling

In August 2019, from each treatment, we collected the 0–10 cm topsoil at five points (arranged in an “X” shape) in each quadrat using a soil auger (diameter, 4 cm) after removing the litter layer on the ground surface and completely mixed the five samples from the same quadrat into one uniform soil sample (Fig. 1). We removed stones and roots from all soil samples collected (i.e., 3 combinations of sand-fixing shrubs × 3 plots/combination × 5 quadrats/plot). The collected soil samples were divided into two parts, one of which was packed in a sterile bag for the analysis of the physical and chemical properties of the soil, and the soil moisture was determined immediately. The other part was passed through a sieve with an aperture of 2 mm, packed into a 15 ml sterile centrifuge tube, quickly put into a dry ice bucket, brought back to the laboratory and stored at − 80 °C for DNA extraction [62].

### Analysis of Soil Physicochemical Properties

Soil moisture was determined by oven drying to a constant mass at 105 ℃. The air-dried soil samples were divided into two parts and passed through soil sieves with apertures of 1 mm and 0.149 mm. The soil pH and electronic conductivity (EC) were determined by a pH meter (PHS-3D, Shanghai Sanxin Instrument, China) and a conductivity meter (DDS-307A, Shanghai Youke Instrument Co., Ltd., China), respectively. The soil total carbon (TC), soil organic carbon (SOC), total nitrogen (TN), available nitrogen (AN), total phosphorus (TP), available phosphorus (AP), total potassium (TK), and available potassium (AK) were measured as described by Bao [2]. Soil C, N, and P and their stoichiometric ratios (SOC/TN, C:N; SOC/TP, C:P; TN/TP, and N:P) were also calculated.

### Soil DNA Extraction, Amplification, and Absolute Quantification Sequencing

The DNA extraction of soil bacteria was performed using a Power Soil™ DNA Isolation Kit (MOBIO Laboratories, Carlsbad, CA, USA). Agarose-gel electrophoresis and Nanodrop 2000 (Thermo Fisher Scientific, USA) were used to detect the integrity and purity of the genomic DNA from the extracted samples, and high-fidelity PCR amplification was performed for the V4-V5 region of the samples in triplicate. It has been reported that the amplified bacterial sequences in the V4-V5 region show the least intragenomic heterogeneity, thus maintaining good specificity and complete database information and minimizing overestimation, making it the best sequencing region for bacteria [8, 75]. Therefore, targeting the V4-V5 regions of 16S rRNA for PCR amplification was conducted using primers 515F (5′-GTGCCAGCMGCCGCGG-3′) and 907R (5′-CCGTCAATTCMTTTRAGTTT-3′) [84]. Sequencing was carried out using the Illumina MiSeq 2 × 250 bp, technically supported by Genesky Biotechnologies Inc., Shanghai, China (201,315) [33]. Over the last few decades, high-throughput sequencing technology has accelerated our understanding of soil microbial diversity and its underlying mechanisms [113]. In this study, a high-throughput absolute quantification sequencing method was employed to obtain an accurate and reliable absolute abundance of soil bacteria because this method can better solve the problem of the relative quantification of microbes in environmental samples while obtaining the exact species composition information, especially with respect to the soil bacterial community and dynamics [99]. The absolute quantification sequencing method eliminates the difficulty in finding specific primers for qPCR validation after relative quantification [73]. In addition, absolute abundance can be used to calculate relative abundance, but the reverse is not true [99].

### Illumina Read Data Processing

To obtain high-quality sequencing data and improve the accuracy of subsequent bioinformatic analysis, the raw sequencing data were quality controlled and filtered. The Trim Galore software was used to remove sequences with a base mass less than 20 at the end of the V4-V5 tag sequences (which contain adapters) and to remove short sequences less than 100 bp in length. FLASH2 software was used to splice paired sequences and remove low-quality sequences (over 90% of base mass below 20). Mothur and UCHIME software were used to find and remove primers, as well as chimeric sequences. Finally, sequences with a similarity of more than 97% were classified as the same OTU, and the resulting representative OTU sequences were used for subsequent species annotation. The proportion of spike-in sequences was marked and counted, and these sequences were removed during subsequent analysis. The standard curve equation was made according to the spike-in sequence of each sample, and the absolute copy number of each OTU in each sample was calculated. Then, the absolute copy number of species in a unit sample was calculated according to the amount of DNA in the sequencing template, the amount of DNA extracted from the sample, and the number of samples used for DNA extraction [84]. There are multiple copies of the 16S gene in a species to ensure survival. In high-throughput sequencing, post-PCR sequencing will amplify this cardinality effect due to the copy number of different bases, resulting in a deviation in the read number for each species. This analysis was based on the rrnDB database (Version V5.6) (https://rrndb.umms.med.umich.edu/). The gene copy number of each OTU was estimated according to the closest relative species, and then, the absolute copy number of OTUs divided by the estimated gene copy number was corrected [72, 92].

### Data Analysis

The changes in soil physicochemical properties among three combinations of shrubs (CaKCoS, CaKCaM, and CoSCaM) were determined based on a one-way analysis of variance (ANOVA) with Duncan’s test by using SPSS 18.0 (IBM Corp., USA). Rarefaction curves, Circos diagrams for the composition of species, Venn diagrams, heatmaps, and comparative diagrams of significantly different phyla were created using the R packages “ggplot2” and “Vegan” (R 4.0.5; https://www.r-project.org/). The alpha diversity (Shannon index) of bacteria was calculated using QIIME2 [3], and a significant difference diagram was drawn using the R package “Vegan.” In the 3 types of leguminous shrub treatments, the top 700 OTUs with significant differences (*P* < 0.05) in phylum-level abundance were filtered out by using SPSS 18.0, and then, the ternary diagram was created using the R package “VCD.” Redundancy analysis (RDA) was performed to elucidate the relationship between bacterial abundance at the phylum level and soil environmental factors using Canonco 5. To demonstrate the relationship between key soil components (C, N, P, and their stoichiometric ratios) and soil bacterial diversity, we constructed a structural equation model (SEM) for all soil samples using the R packages “lavaan,” “haven,” “Hmisc,” and “semPlot” [16]. All of the images described above were processed by Adobe Illustrator CS6, and the significant differences are expressed as *P* < 0.05, *P* < 0.01, and *P* < 0.001.

## Results

### Effects of Leguminous Shrubs on Soil Bacterial Diversity in Sandy Land

**Fig. 2:**
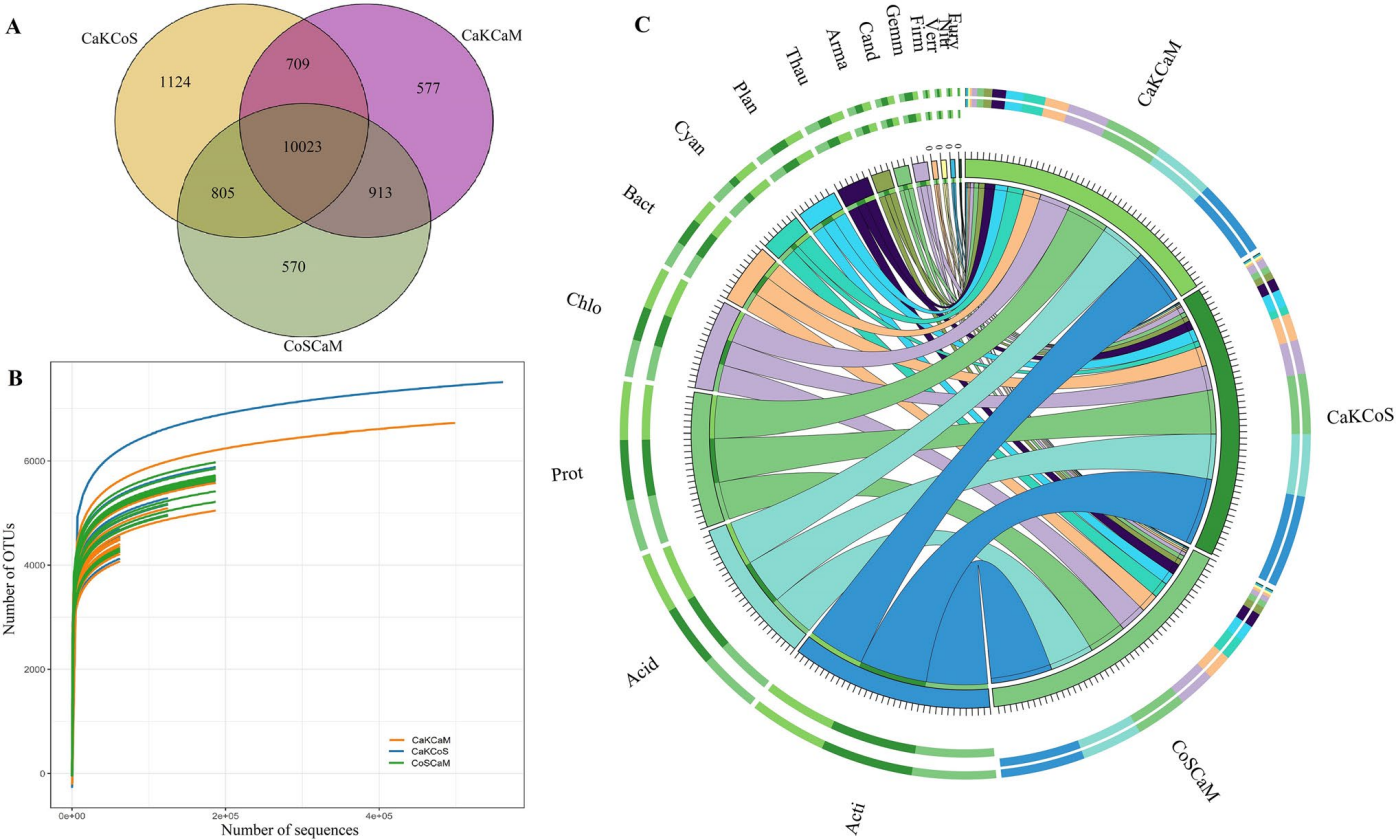
Effects of three combinations of shrubs on soil bacterial communities (**A** Venn diagram; **B** Rarefaction curve; and **C** Circos plots showing the abundance at the phylum level. All phyla are abbreviated by the first four letters of their names. Acti, Acid, Prot, Chlo, Bact, Cyan, Plan, Thau, Arma, Gemm, Cand, Firm, Verr, Nitr, and Eury are abbreviated forms of Actinobacteria, Acidobacteria, Proteobacteria, Chloroflexi, Bacteroidetes, Cyanobacteria/Chloroplast, Planctomycetes, Thaumarchaeota, Armatimonadetes, Gemmatimonadetes, candidate_division_WPS-1, Firmicutes, Verrucomicrobia, Nitrospirae, and Euryarchaeota*,* respectively). CaKCoS, CaKCaM, and CoSCaM represent the mixture of *C. korshinskii* and *C. scoparium*, the mixture of *C. korshinskii* and *C. mongolicum*, and the mixture of *C. scoparium* and *C. mongolicum*, respectively

**Fig. 3:**
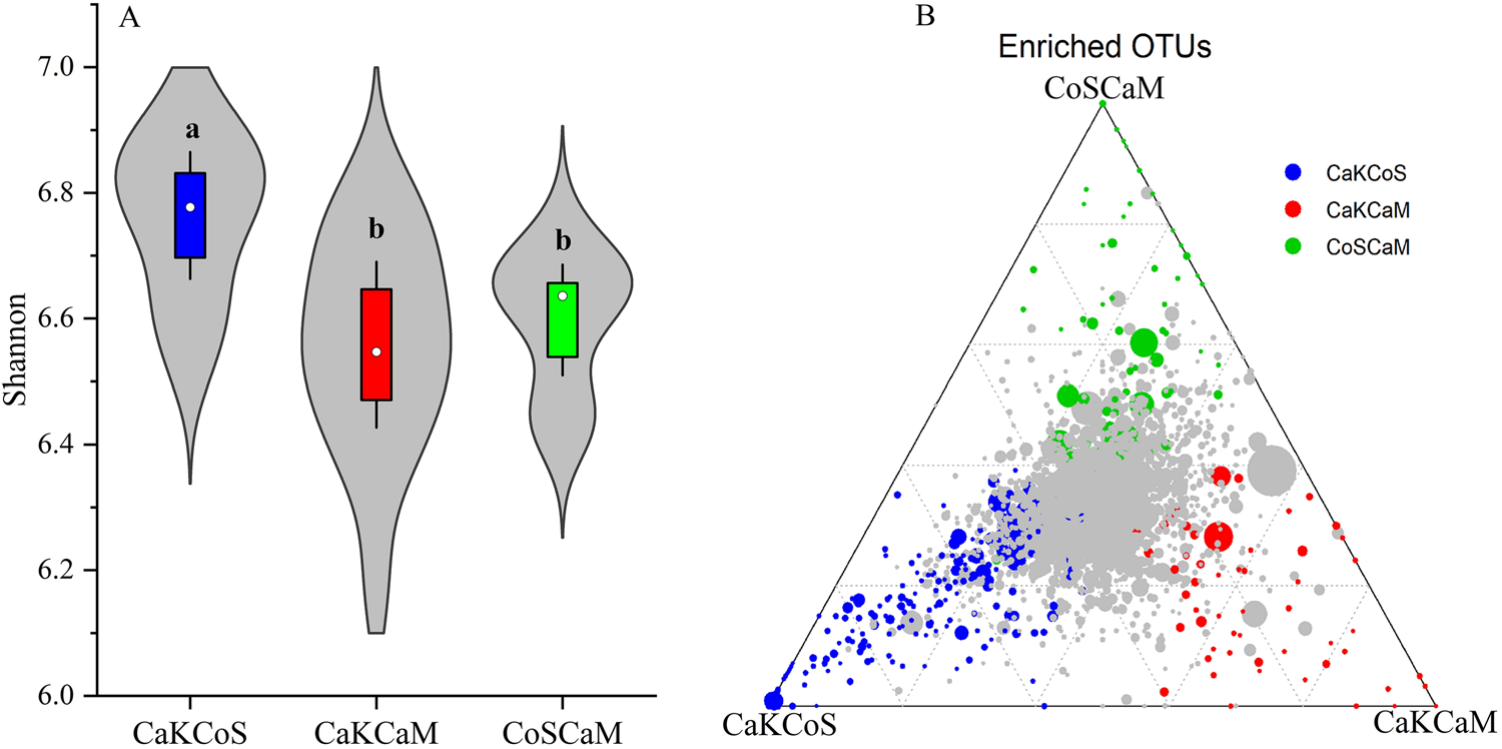
Comparison of soil bacterial diversity in different combinations of sand-fixing shrubs (**A** Shannon index; **B** Ternary diagram. Solid dots represent significantly different OTUs (*P* < 0.05), the size of the dots indicates the abundance of the OTUs, and gray dots represent OTUs that are not significantly different). CaKCoS, CaKCaM, and CoSCaM represent the mixture of *C. korshinskii* and *C. scoparium*, the mixture of *C. korshinskii* and *C. mongolicum*, and the mixture of *C. scoparium* and *C. mongolicum*, respectively

After quality screening, read removal, and statistical analysis, we obtained 10,137,510 reads from 45 soil samples by 16S rRNA gene sequencing. The number of clean reads per sample ranged from 126,903 to 762,429, and an average of 225,278 clean reads was isolated from each soil sample (Table S1). The Venn diagram showed that 10,023 OTUs were shared by the three combinations of shrubs from the absolute copy number of genes per gram of soil, and the CaKCoS treatment had 1124 unique OTUs, which was the highest among treatments (Fig. 2A). The rarefaction curve indicated that the sequences of all samples covered the diversity of bacterial populations across the three combinations of shrubs (Fig. 2B). The dominant phyla identified for absolute quantitative 16S rRNA sequencing from the three combinations of shrubs included Actinobacteria (20.57–25.4%), Acidobacteria (16.4–16.89%), Proteobacteria (13.92–16.77%), Chloroflexi (9.03–10.93%), Bacteroidetes (6.45–7.47%), Cyanobacteria/Chloroplast (2.07–6.53%), Planctomycetes (4.55–4.86%), Thaumarchaeota (3.27–3.82%), Gemmatimonadetes (1.39–2.29%), Armatimonadetes (1.96–2.31%), and candidate_division_WPS-1 (1.65–1.8%) (Fig. 2C). The Shannon index in CaKCoS was significantly higher than that in CaKCaM and CoSCaM (*P* < 0.01, Fig. 3A). The ternary diagram showed a more diverse OTU enrichment in CaKCoS than in CaKCaM and CoSCaM (Fig. 3B). These results suggested that the diversity of soil bacteria was strongly associated with the sand-fixing combination of the two leguminous shrubs (*C. korshinskii* × *C. scoparium*) rather than that with one leguminous shrub.

**Fig. 4:**
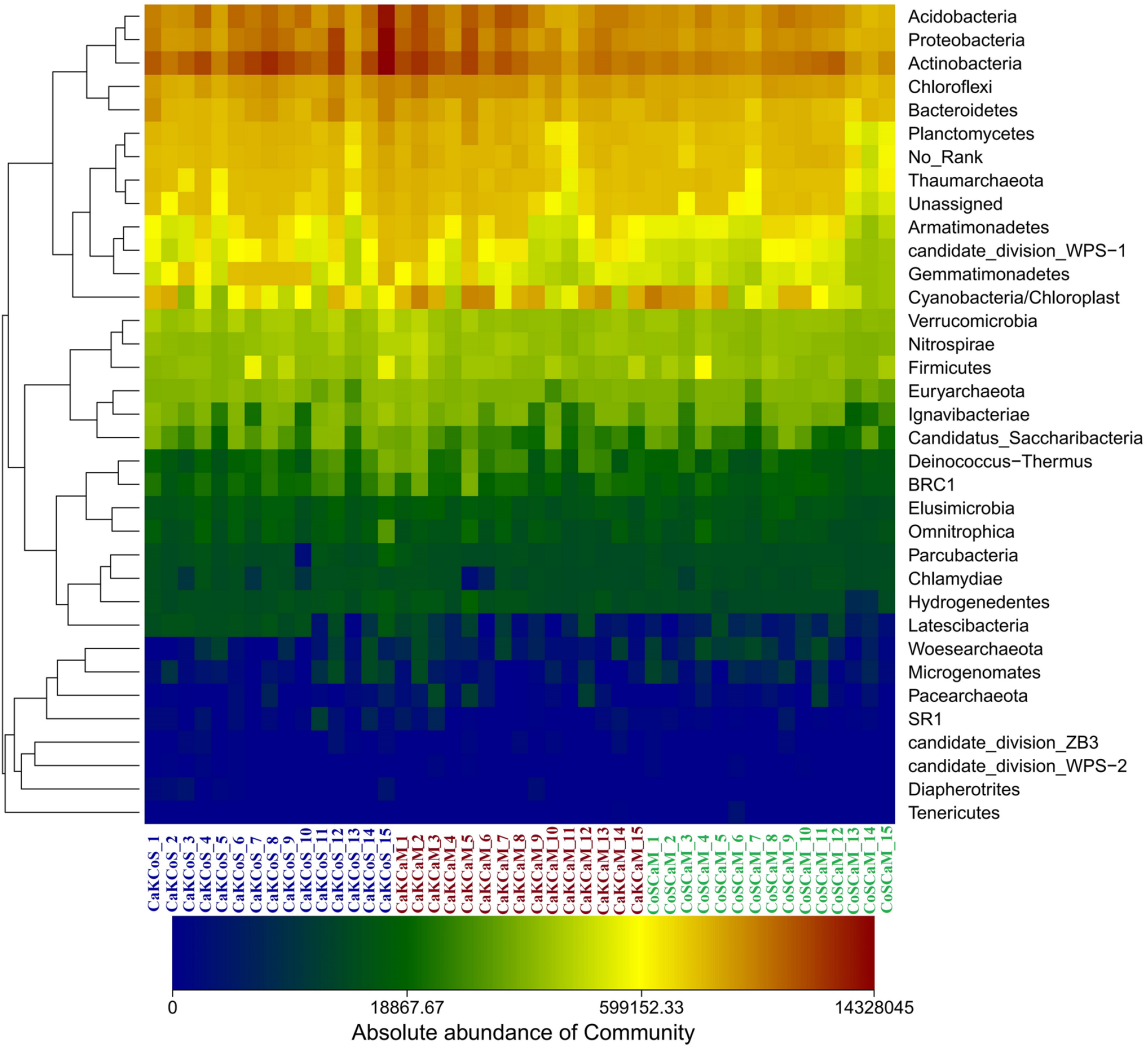
Heatmap and hierarchical cluster diagram based on Euclidean distance of the abundant phyla of the soil bacteria in different combinations of sand-fixing shrubs. The transition from blue to red reflects the gradual change in abundance from low to high. CaKCoS, CaKCaM, and CoSCaM represent the mixture of *C. korshinskii* and *C. scoparium*, the mixture of *C. korshinskii* and *C. mongolicum*, and the mixture of *C. scoparium* and *C. mongolicum*, respectively

A heatmap was constructed to show the phylum-level abundance of bacteria among the three combinations of shrubs (Fig. 4). This result indicated that phyla clustered into a clade according to their abundance. Crucially, the abundance of the most dominant phyla was higher in CaKCoS and CaKCaM than in CoSCaM, while the abundance of Cyanobacteria was higher in CaKCaM and CoSCaM than in CaKCoS. Among these dominant phyla, Actinobacteria, Acidobacteria, Proteobacteria, Chloroflexi, Planctomycetes, Thaumarchaeota, Armatimonadetes, candidate_division_WPS-1, Verrucomicrobia, BRC1, and Nitrospirae were significantly more abundant in CaKCoS and CaKCaM than in CoSCaM (*P* ≤ 0.05) (Fig. S1).

### Relationships Between Bacterial Communities and Soil Physicochemical Properties

**Fig. 5:**
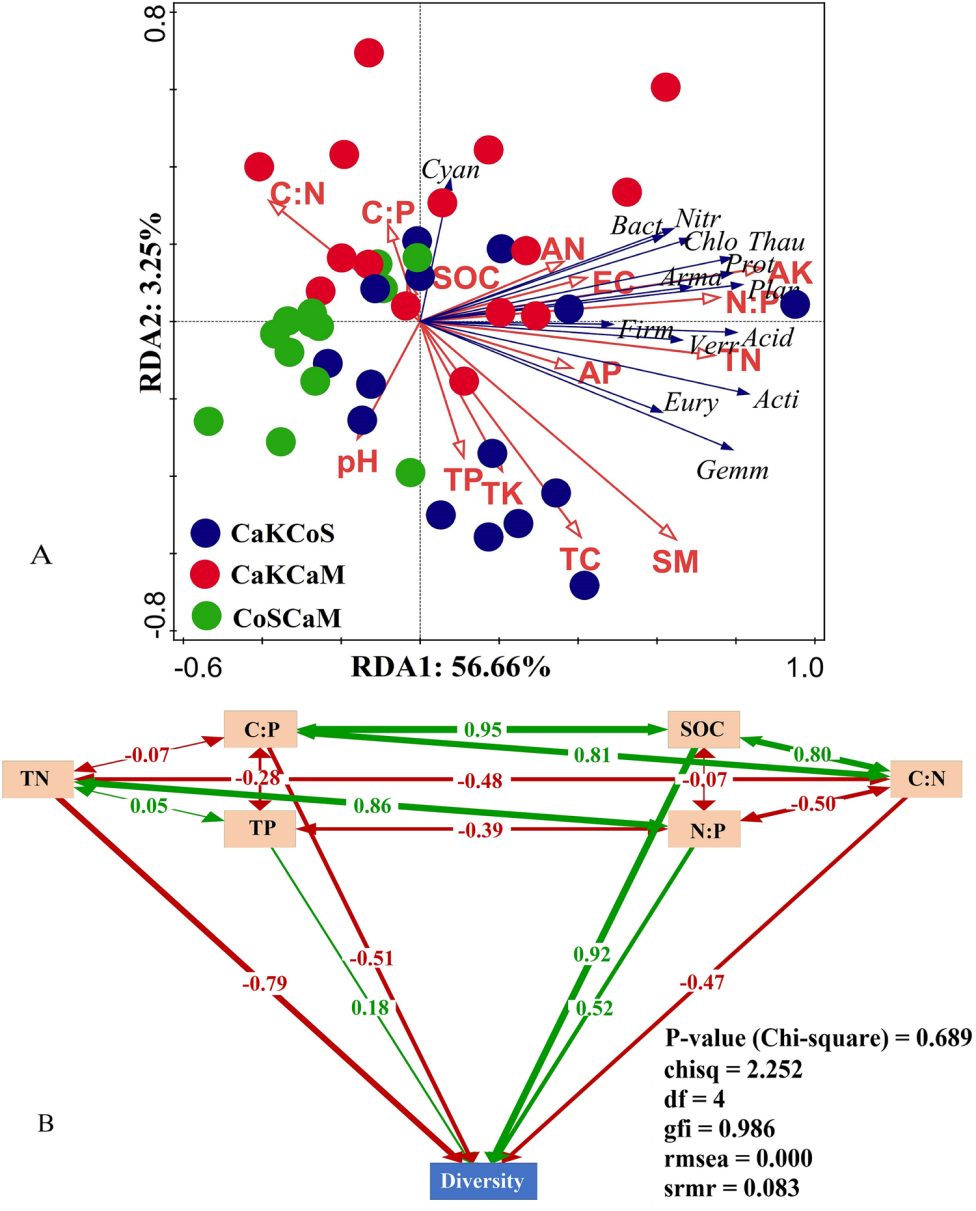
Relationship between soil bacterial diversity and soil physicochemical properties (**A** Redundancy analysis indicating the relationship between the bacterial abundance of dominant phyla (blue arrows), edaphic factors (red arrows), and sample plot (solid dot with different colors). All species at the phylum level are abbreviated as the first four letters of their names; **B** Path diagram of the structural equation modeling (SEM) for all samples. Positive correlations are indicated by green arrows, and negative correlations are indicated by red arrows in SEM)

RDA indicated that the majority of the soil factors were significantly positively correlated with the abundance of the dominant phyla, including Actinobacteria, Acidobacteria, Proteobacteria, Chloroflexi, Planctomycetes, Thaumarchaeota, and Armatimonadetes. Canonical RDA1 and RDA2 accounted for 56.66% and 2.35% of the total variation, respectively (Fig. 5A). TN, N:P, SM, and AP significantly affected the abundance of bacterial phyla in the soil. Considering the relationship with the sample plots, the promotion mainly occurred in CaKCoS- and CaKCaM-containing *C. korshinskii*.

The SEM results showed that the model could well explain the relationship between soil bacterial diversity and soil C, N, and P nutrients (Fig. 5B). There were significant SEM paths based on all samples (P(χ^2^) = 0.689, chisq = 2.252, df = 4, gfi = 0.986, rmsea = 0.000, srmr = 0.083). SOC (0.92), N:P (0.52), and TP (0.18) were positively correlated with the bacterial Shannon index, which plays a crucial role in shaping bacterial diversity. However, TN (− 0.79), C:P (− 0.51), and C:N (− 0.47) were negatively correlated with soil bacterial diversity. Collectively, our study emphasizes that the combination of sand-fixing shrubs shapes bacterial diversity by changing the soil physical and chemical properties. Interestingly, this diversity occurred in the combination containing two leguminous shrubs but was not found in the combination containing one leguminous shrub.

### Comparison of Soil Physical and Chemical Properties Among Different Combinations of Sand-Fixing Shrubs

The soil physical and chemical properties at a depth of 0–10 cm significantly differed among the three combinations of shrubs, as shown in Table 1 (*P* < 0.05). The CaKCoS and CaKCaM treatments had significantly higher SOC, TN, AK, EC, N:P, and C:P, while the contents of SM, TC, TK, and AP were all higher in CaKCoS than in CaKCaM and CoSCaM. Briefly, these results reinforced that the soil fertility was effectively enhanced in the sand-fixing combination containing two leguminous shrubs than in that containing one leguminous shrub, indicating that the combination of legumes is more beneficial for restoring sandy land ecosystems.

**Table 1:** Soil physicochemical properties in different combinations of sand-fixing shrubs

Shrub type	CaKCoS	CaKCaM	CoSCaM
SM %	0.65 ± 0.06a	0.41 ± 0.03b	0.35 ± 0.04b
pH	8.63 ± 0.03a	8.54 ± 0.03b	8.66 ± 0.02a
EC	70.43 ± 1.70a	74.06 ± 3.30a	61.15 ± 1.09b
TC g·kg^−1^	6.73 ± 0.40a	5.59 ± 0.21b	5.83 ± 0.25b
SOC g·kg^−1^	1.71 ± 0.23ab	2.21 ± 0.34a	1.33 ± 0.14b
AK mg·kg^−1^	98.33 ± 4.70a	96.55 ± 5.28a	81.71 ± 3.22b
TK g·kg^−1^	28.05 ± 0.83a	26.24 ± 0.35b	26.12 ± 0.41b
AP mg·kg^−1^	1.09 ± 0.10a	0.78 ± 0.07b	0.85 ± 0.07b
TP g·kg^−1^	0.22 ± 0.01a	0.21 ± 0.01a	0.23 ± 0.02a
AN mg·kg^−1^	1.96 ± 0.12a	2.24 ± 0.23a	1.99 ± 0.16a
TN g·kg^−1^	0.0962 ± 0.01a	0.0896 ± 0.01a	0.0532 ± 0.01b
C:N	19.65 ± 2.78a	37.57 ± 9.48a	26.02 ± 3.10a
C:P	7.79 ± 0.90ab	11.11 ± 1.78a	5.93 ± 0.63b
N:P	0.45 ± 0.04a	0.42 ± 0.04a	0.25 ± 0.02b

Values are means ± SEs (n = 43). Different letters represent significant differences (*P* < 0.05) among different combinations of sand-fixing shrubs according to one-way ANOVA followed by Duncan’s test. Soil factors are abbreviated as follows: *SM*, soil moisture; *TC*, total carbon; *SOC*, soil organic carbon; *TN*, total nitrogen; *AN*, available nitrogen; *TP*, total phosphorus; *AP*, available phosphorus; *TK*, total potassium; *AK*, available potassium; *EC*, electronic conductivity; C:N, SOC/TN; C:P, SOC/TP; and N:P, TN/TP. CaKCoS, CaKCaM, and CoSCaM represent the mixture of *C. korshinskii* and *C. scoparium*, the mixture of *C. korshinskii* and *C. mongolicum*, and the mixture of *C. scoparium* and *C. mongolicum*, respectively.

## Discussion

### The Restoration of Sandy Land with Leguminous Shrubs Could Effectively Drive Soil Bacterial Diversity

The novelty of this study was that we applied absolute quantification sequencing of 16S rRNA genes to accurately determine the changes in soil bacterial abundance in sand-fixing shrub combinations involving leguminous shrubs. In this study, we found that the dominant phyla were sequenced from the three combinations of shrub plantations (Fig. 2). After fixing the dunes with straw checkerboards, the combination of planting two leguminous shrubs (CaKCoS) significantly increased the diversity of soil bacteria and enriched the significantly different OTUs (Fig. 3). This result confirmed our hypothesis and provided a conceptual framework that the combination of leguminous shrubs can drive soil bacterial diversity during the process of desertification reversal. A large number of studies have shown that vegetation restoration involving legumes can effectively promote the α-diversity of soil bacteria, such as drought-tolerant legume shrub afforestation by using *C. korshinskii* [108], *R. pseudoacacia*, or *C. korshinskii* leguminous forestland [63, 64, 97], and 32-year-old *C. microphylla* plantations [5, 110], which supported the results of this study. This result might be because legumes enrich soil nutrients through N fixation and litter input and provide resource stores for bacterial communities to multiply in desert areas [100, 116]. *C. korshinskii*, *R. pseudoacacia*, and *C. microphylla* are typical leguminous shrubs used in forestation, and the dominant bacterial phyla found in these shrublands were almost consistent with the sequencing results in our study. The key factor driving soil bacterial diversity lay in the combination of species, especially legumes.

Another explanation could be that soil microbial diversity is closely related to soil biological crusts in dryland ecosystems, and litter that was produced by planting xerophytic shrubs to stabilize the moving dunes contributes to creating a suitable habitat for the occurrence of biological crusts [43]. Litter is retained on the soil surface for a long time and gradually forms the litter crust, which is the main contributor of C resources and plays an important role in maintaining the function of the soil ecosystem. The soil bacterial community and nutrient enrichment documented in Liu et al. [42] were closely related to the increase in SOC and TC, which were associated with litter crusts, and the diversity of bacteria in the litter crust was also significantly higher than that in bare soil. The key factor is that litter crusts are involved in nutrient regulation in the plant‒soil-microbe continuum and promote the accumulation of C and N on the soil surface [32, 42]. Studies have shown that litter crust depth is closely related to soil microbial diversity [41]. Li et al. [37] reported that the litter quality and soil enzyme activity of a *C. korshinskii* plantation were higher than those of a *Platycladus orientalis* plantation, indicating that legumes were the most effective means of afforestation in drylands. Therefore, the improvement of soil nutrients caused by litter crusts was likely to be a pathway for the enrichment of soil bacterial diversity.

Differences in plant functional composition contribute to differences in microbial community composition, which is a common indication of ecosystem functioning [11, 26]. In our study, the sand-fixing combination of two leguminous shrubs effectively increased the abundance of dominant soil phyla, including Actinobacteria, Acidobacteria, Proteobacteria, Chloroflexi, Planctomycetes, Thaumarchaeota, and Armatimonadetes (Fig. 4); among these phyla, the most obvious changes in order were found for Actinobacteria, Gemmatimonadetes, Proteobacteria, Chloroflexi, candidate_division_WPS-1, and BRC1 (Fig. S1). The cycling and balance of soil C and N are closely related to these bacterial phyla [21]. Studies have shown that Actinobacteria, as a copiotrophic phylum, had a high content in the soil of vegetation restoration areas, while Acidobacteria, as an oligotrophic phylum, was commonly abundant in cropland [28]. In general, copiotrophic and oligotrophic bacteria were taken as indicators for assessing the soil environment [38]. Actinobacteria is a typical phylum of gram-positive bacteria that helps to decompose dead organisms in the soil, promotes material circulation, produces antibiotics, and makes a great contribution to human health [67]. Acidobacteria are widely distributed bacteria with high variation and high abundance in low organic C environments [34]. Proteobacteria is a phylum of gram-negative bacteria, and some α- or β-Proteobacteria can also grow in low nutrient environments, inducing N fixation and forming symbionts with plants [56, 60]. Proteobacteria was one of the most important phyla in the leguminous shrub plantation in the present research. Ren et al. [63] found that soil bacterial diversity significantly increased when afforestation with *C. korshinskii* was compared with abandoned land and that afforestation effectively increased the abundance and dominance of Proteobacteria. These results indicate that the dominance of the N-fixing groups in the soil bacteria was closely related to the improvement of soil N with the introduction of legumes, which was consistent with the results of our study. Many studies have confirmed that the mineralization rate of soil C and the cycling of N could be predicted by the abundance of Proteobacteria, Acidobacteria, Bacteroidetes, and Actinobacteria; in particular, almost half of the Rhizobiales in Proteobacteria were directly associated with N fixation [21, 80]. In a study of the rhizobia of the leguminous plant *Dalbergia*, among 68 isolated bacteria, 65 belonged to Proteobacteria, especially *Burkholderia*, which can significantly induce the formation of nodules in the roots of leguminous plants [60]. Past studies also showed that the dominant phyla found in the present study, including Proteobacteria, Actinobacteria, and Bacteroidetes, were the main group in the rhizosphere of *Hedysarum* during afforestation with two leguminous shrubs [116]. Proteobacteria have been proven to be closely related to soil C availability [21],the input of C sources affected the content of soil nutrients, changed the C:P and N:P ratios, and ultimately influenced the change in microbial composition. Additionally, with the increase in forestation years of *C. korshinskii*, Proteobacteria in the soil became increasingly abundant, while Actinobacteria decreased correspondingly [108]. In the leguminous *C. korshinskii* and *R. pseudoacacia* plantations, the abundance of Actinobacteria was higher than that in grassland [95], which was similar to the results of this study. A large number of N-fixing microorganisms enriched the soil of the *C. korshinskii* plantation [98], which effectively increased the soil N. Therefore, the significant increase in the dominant bacteria in leguminous shrub combinations might also be related to the increase in N:P and C content in the present study.

Cyanobacteria are photosynthetic bacteria that play an important role when subjected to environmental stress. Biological soil crusts with Cyanobacteria can be isolated in many desert systems [78, 90]. After years of sand fixation with straw checkerboards combined with different shrubs, it was found that the abundance of Cyanobacteria was higher in the combination containing one leguminous and one nonleguminous shrub (CaKCaM) (Fig. 4). Strong habitat stress on the soil surface, especially water stress, can induce Cyanobacteria colonization, but Cyanobacteria must be equipped with mechanisms to protect themselves and avoiding high light intensities. More importantly, moderate fluctuation in environmental water creates conditions for Cyanobacteria to thrive, while continuous water conditions may lead to some cyanobacterial deaths [68, 90]. Cyanobacteria do not have the ability to mobilize water from deep soil layers and can only rely on the amount or frequency of precipitation to perform ecological functions such as N fixation [57], which might be related to the moderate SM content (Table 1) in the leguminous and nonleguminous shrub combination in this study. A previous study confirmed that Cyanobacteria in the bare substrate biocrusts of mountain vegetation types in the northern Urals were specific to different plant communities [52], which suggests that our future research needs to pay more attention to the effects of different sand-fixing plant communities on Cyanobacteria. Another possibility is that the sand-fixing combination of two leguminous shrubs inputs functional litter to the soil [71], leading to a significantly higher leaf C, N, P, and K content and a higher N:P ratio than those of nonleguminous plants [25], which further leads to a higher soil bacterial diversity and increases the abundance of bacterial phyla, such as Actinobacteria, Proteobacteria, Chloroflexi, Acidobacteria, and Bacteroidetes, in the soil instead of Cyanobacteria. This result indicates that leguminous plants had a strong competitiveness in leaf functional traits and changed the quality and properties of the litter.

### The Improvement in Soil Properties Contributed Greatly to the Bacterial Diversity in Different Combinations of Sand-Fixing Shrubs

Our results showed that higher soil bacterial diversity was caused by improvements in soil physicochemical properties, which was attributed to the combination of leguminous shrubs used for sand fixation and afforestation. This may be due to the legume‒legume combinations triggering different N-fixing capacities and leading to the functional differentiation of soil nutrients [15, 69]. This result indicated that bacterial diversity was promoted by SOC, N:P, and TP but inhibited by TN, C:N, and C:P (Fig. 5B), and the abundance of dominant phyla was promoted by TN, AK, N:P, SM, and AP (Fig. 5A). This result might be because the combination of two leguminous shrubs produced litter, which initiated a considerable C pool, and the bacterial community, which was dominated by Proteobacteria, Actinobacteria, and Acidobacteria, depended crucially on the litter characteristics of specific plants [44], especially the high yield and quality of leguminous litter [29, 37], and its leaf N fixation traits [12]. Therefore, it has been documented that the properties of litter produced by legumes are key to the contribution of soil bacterial diversity. Similar studies have been reported, for example, in restoration patterns of legumes (*R. pseudoacacia*, *C. korshinskii*), litter characteristics significantly increased the Chao1 index [95] and community composition [94] of soil bacteria. The litter layer was the resource store of the ecosystem, containing nutrients such as C, N, and phosphorus (P), as well as other matter, which was driven by the microbial community [9], and litter quality was the crucial factor in promoting bacterial diversity in the topsoil layer [35].

The accumulation of SOC from leguminous shrub litter increased the metabolic function of Actinobacteria, while Acidobacteria was abundant in lower soil resources [38, 94]. Xu et al. [96] found that leguminous shrubs could significantly improve the Shannon and Chao1 indices of soil microorganisms, which were also significantly affected by SOC [100]. These findings were similar to our results. Thus, we suggest that C accumulation induced by leguminous litter might be a more favorable sand-fixing strategy that results in higher bacterial diversity. Recent studies supported that litter crusts positively promote the input of SOC and TN, which was strongly related to the bacterial community [42]. Viruel et al. [81] also found that SOC was one of the factors that determined the α-diversity of bacteria, which is consistent with our results, but the promoting effect of TN was contrary to that in the present study. The soil TN content effectively increased the abundance of bacteria (Fig. 5A), but TN and the C:N and C:P ratios decreased the diversity (Fig. 5B), which was different from some previous studies [62, 94]. Liu et al. [38] reported that *R. pseudoacacia*, a typical legume, significantly improved soil bacterial abundance when used alone, and this result was similar to those of our study. In summary, we proposed that the bacterial diversity might ultimately be due to the increased rate of C mineralization rather than N mineralization [76]. In contrast, in the desert oligotrophic environment, soil N was likely to stimulate the abundance of specific phyla but not the conditions that triggered increased diversity.

In this study, the N:P ratio could promote soil bacterial diversity under the combination of two leguminous shrubs. For example, in the Loess Plateau of China, the longer that *C. korshinskii* was planted, the more correlated the soil microbial diversity and dominant phyla abundance were with the N:P and C:P ratios, especially the N:P ratio [108]. The results suggested that the correlation between the N:P ratio and microbial diversity had a more useful effect on the changes in the soil microbial community than the C:N and C:P ratios in the leguminous plantations of *C. korshinskii* and *R. pseudoacacia* [61, 64], which was consistent with the results of this study. The negative correlation between the C:P ratio and bacterial diversity might be because N-fixing microbes increased continuously after long-term afforestation with leguminous shrubs in sandy land, resulting in imbalances in the soil C, N, and P. The soil bacterial diversity was closely related to P accumulation [14, 94]. The evidence shows that bacterial diversity peaks under the conditions of high P availability, soil organic matter reduction, and low C:P and C:N [14], and these soil conditions tend to accelerate nutrient cycling [13] and increase bacterial diversity by reducing fungal diversity [80]. Thus, these results supported our argument (Fig. 5).

### The Restoration of Sandy Land with Leguminous Shrubs Could Effectively Improve the Soil Physical and Chemical Properties

In the present study, the combination of leguminous shrubs in sand fixation could effectively improve the soil properties (Table 1). Before 2001, our sampling plot suffered from severe disturbances due to moving dunes. After the long-term sand fixing practice was implemented, the combination of xerophytic shrubs gradually planted in the checkerboards stopped dune movement, and the physical and chemical properties of the soil gradually improved. Interestingly, we found that the combination of two leguminous shrubs (CaKCoS) significantly increased the soil physicochemical properties, such as SM, TC, TK, and AP (Table 1). Previous studies have confirmed that the long-term afforestation of *C. korshinskii* significantly promoted the accumulation of soil TC, SOC, TN, AK, and AN contents, which strongly supported our study results [37]. This was most likely related to the litter quality and soil enzymatic activity of legumes [30], suggesting that the use of specific species is the key to the improvement of soil properties. This might be driven by the improvement of the root exudates and the decomposition of the litter residues [53], and it might also be due to the dual N-fixation by the combination of legumes [49, 89]. The key factor is that legumes are special functional groups with higher productivity that feed back to the soil and promote soil fertility [23]. Most of the contributions to the soil surface layer come from N fixation and litter properties [100, 116]. The N-fixing function of leguminous leaves provides the soil with rich litter C and N resources [12, 15, 50], thus driving bacterial diversity by promoting soil fertility, which supported the hypothesis of this study. The functional traits and symbiotic systems of legumes explained the excellent performance of leguminous shrubs in soil improvement [94]; for example, the litter production and quality of arboreal legumes (*Gliricidia sepium*) (Herrera et al., 2020) and decomposition rete [31, 71] promoted soil nutrient cycling. The asymbiotic leaf N-fixation traits also contribute significantly to soil N and C accumulation by mediating litter properties [50]. We also found that the highest SOC and AN contents were in CaKCaM, which is consistent with Zhou et al. [114] and might be due to the abundance of Cyanobacteria in this combination during sand fixation.

In general, the long-term afforestation of xerophytic shrubs could release abundant nutrients to the soil, especially the combination of leguminous shrubs, which benefits from the unique functional traits of legume coupling. In the *C. microphylla* nebkha, which is a leguminous shrub, the enrichment rates of soil organic matter (SOM), AN, and AP were higher than those in nonlegumes (*Atraphaxis manshurica* and *Salix gordejevii*) [4]. Our previous studies have confirmed that soil C, N, and K are the key factors in vegetation restoration in desertification reversal areas [59], this result was well verified in our sample plots in this study. A meta-analysis by Gao and Huang [24] showed that the Three-North Shelter Forest construction project could significantly improve the soil AK content (72.93%) in the 0–20 cm soil layer of the Loess Plateau, especially in leguminous forests of *R. pseudoacacia* and *C. korshinskii*, which was similar to the result of higher potassium in this study. Crucially, our results suggested that the contents of SM, TC, TK, and AP were significantly higher in the combination of two leguminous shrubs than in the other treatments (Table 1). SM is the key driving factor of afforestation success in desert areas. *Caragana* shows strong water utilization adaptability in long-term afforestation practices [112], for instance, the water use capacity of *Hedysarun fruticosum* gradually yielded to that of *C. microphylla* [27]. A study confirmed that *C. korshinskii* had a high dependence on water content in the deep soil layer (80–100 cm) during the drought period [112], and the increase in water content in the surface layer (0–10 cm) in this study might be closely related to the nursing effect [39] of the canopy formed by the intermixing of *C. korshinskii* and *C. scoparium*. Under this shrub canopy, a higher content of fine particle fractions with strong water-holding capacity was formed [106], and the combination of these two shrubs (CaKCoS) gradually contributed to the formation of C, N, and P stores, called “resource islands” [1, 101]. Zhang et al. [109] found that *C. korshinskii* had a stronger effect on enhancing the preservation of soil fine particle composition than natural grassland restoration, and the SM content in the 0–100 cm soil layer was significantly positively correlated with the fine particle fraction (silt and clay). A large number of studies have confirmed that the content of soil nutrients is the highest in fine particles of silt or clay [51, 83, 86, 109].

In conclusion, our study demonstrated a conceptual framework in which the combination of leguminous shrubs enhanced soil bacterial diversity by improving soil physicochemical properties, which are key factors in the accumulation of soil C, N, and P. This study provides a theoretical basis for effectively promoting ecosystem functioning in the struggle to fix sandy dunes and provides a scientific basis for the practice of vegetation recovery in arid and semiarid regions.

## Conclusion

In this study, we took three combinations of sand-fixing shrubs as research objects. The combination of two leguminous shrubs (CaKCoS) and combinations of one leguminous and one nonleguminous shrub (CaKCaM and CoSCaM) were analyzed to explore the soil physicochemical properties, bacterial diversity, and community composition, as well as their driving factors, based on high-throughput absolute quantification 16S rRNA sequencing.

**Fig. 6:**
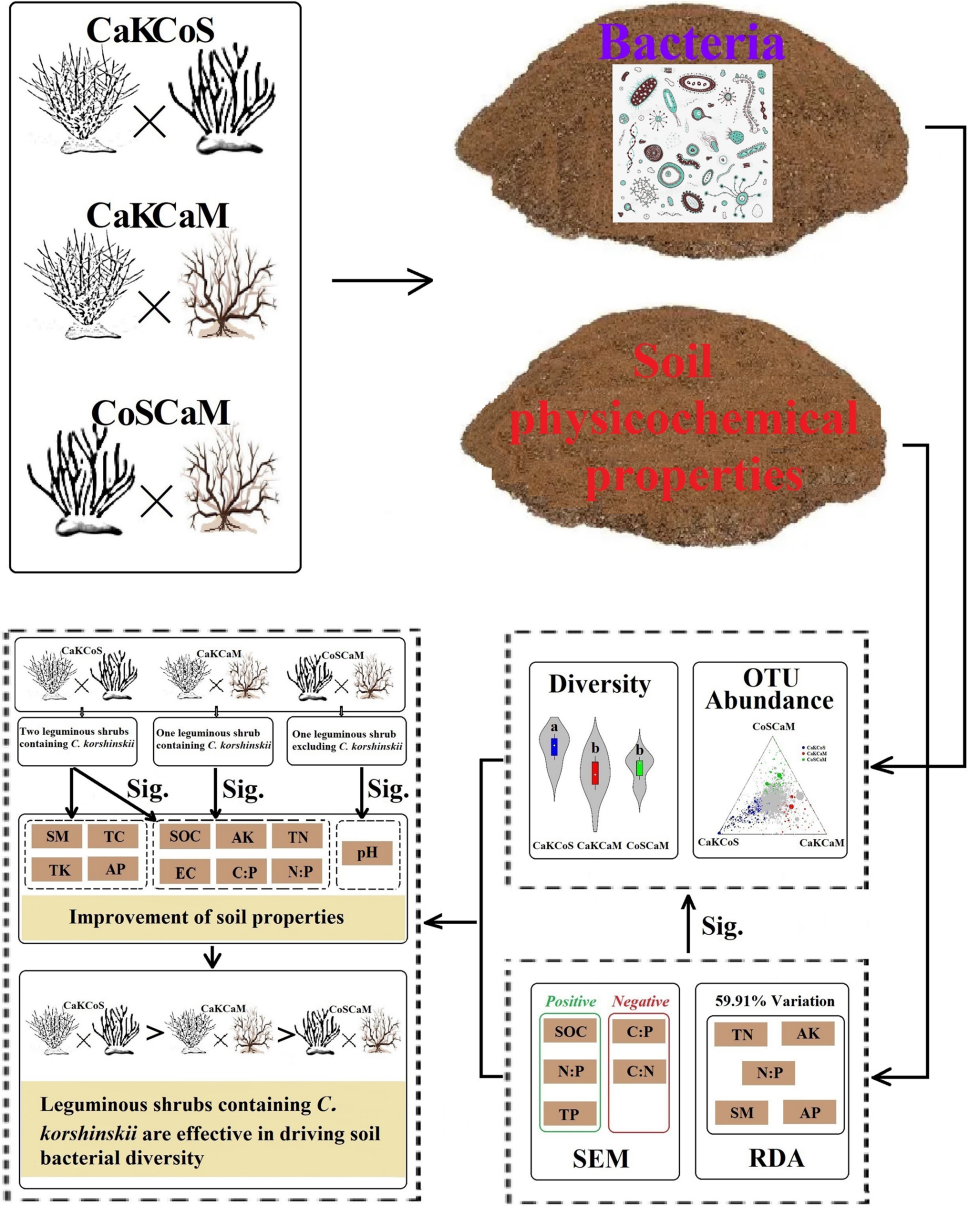
The guiding principle diagram indicates the general conclusions of this study (Sig. represents the significant influence on the factor the arrow is pointing to; CaKCoS, CaKCaM, and CoSCaM represent a mixture of *C. korshinskii* and *C. scoparium*, mixture of *C. korshinskii* and *C. mongolicum*, and mixture of *C. scoparium* and *C. mongolicum*, respectively)

Our results suggested that the combination of two leguminous shrubs (CaKCoS) significantly improved soil physicochemical properties and soil bacterial diversity (Fig. 6). The abundance of the dominant phyla in the bacterial community composition of CaKCoS was significantly higher than that of CaKCaM and CoSCaM, the dominant bacterial phyla of which were mainly *Actinobacteria*, *Acidobacteria*, *Proteobacteria*, *Chloroflexi*, *Planctomycetes*, and *Thaumarchaeota*. The RDA indicated that the majority of soil properties (TN, AK, N:P, SM, and AP) were important soil environmental factors affecting the abundance of the dominant phyla, and canonical RDA1 and RDA2 accounted for 56.66% and 2.35% of the total variation, respectively. SEM revealed that soil bacterial diversity was positively affected by SOC, N:P, and TP but negatively affected by C:P and C:N. Among these factors, SOC and N:P were the crucial factors influencing soil bacterial diversity, and soil P was probably the main limiting factor. This study elucidates that a combination of leguminous shrubs is the crucial driving factor for soil bacterial diversity and is most likely attributable to the litter properties of legumes.

Overall, our results highlight a conceptual framework for sand-fixing afforestation and vegetation recovery in sandy land, that is, the combination of leguminous shrubs, which effectively improved soil properties, drove soil bacterial diversity, and maintained ecosystem functioning and balance during desertification reversal.

## Supplementary Information

Below is the link to the electronic supplementary material.

Supplementary file1 (JPG 413 KB)
