# Supplementary figures and images for: High-Throughput Absolute Quantification Sequencing Reveals that a Combination of Leguminous Shrubs Is Effective in Driving Soil Bacterial Diversity During the Process of Desertification Reversal
Source: Microb Ecol. 2022 Dec 10;86(2):1145–63. doi: 10.1007/s00248-022-02151-0 (PMC10335958; doi:10.1007/s00248-022-02151-0)

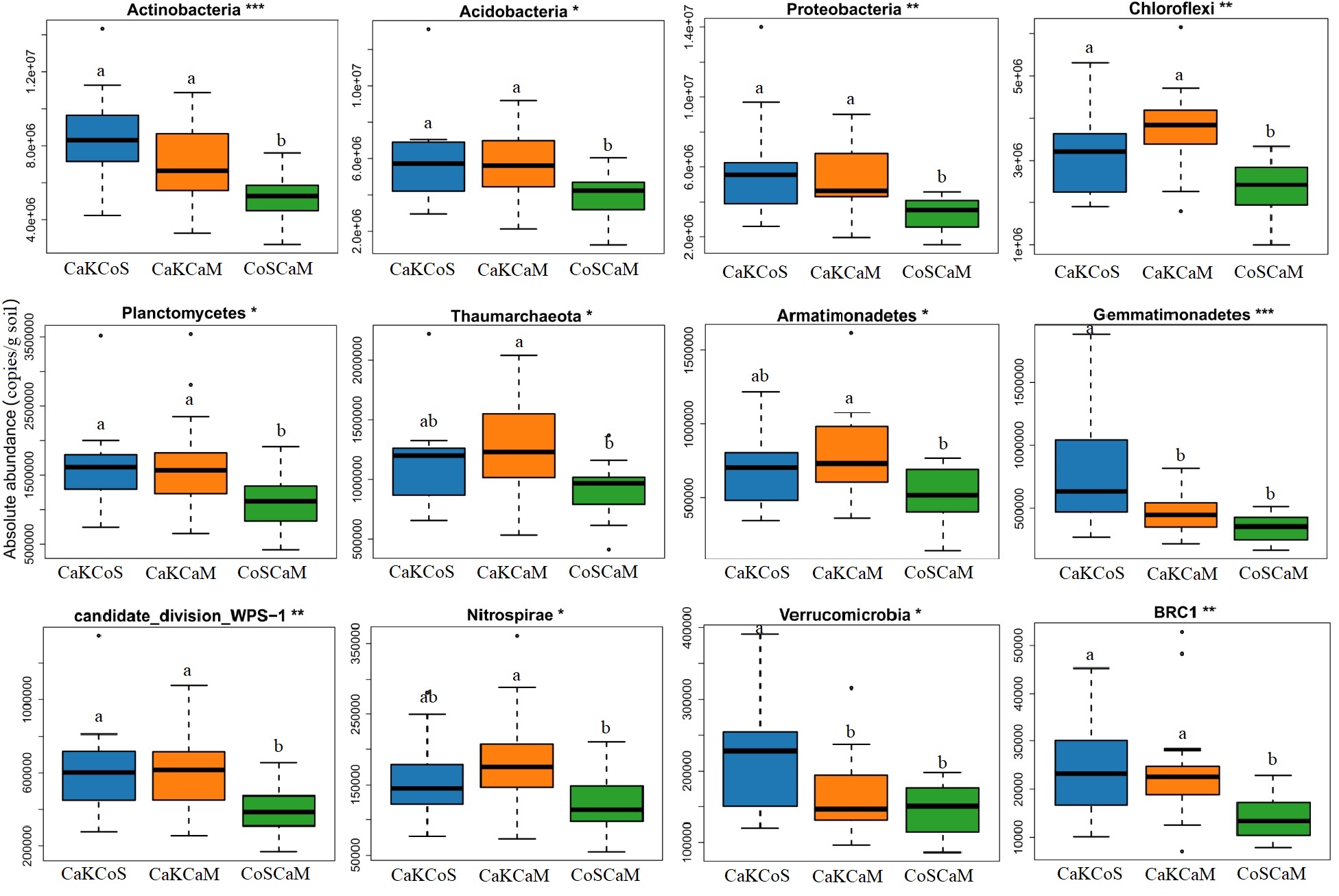

Supplement: Supplementary file 1 — Supplementary file1 (JPG 413 KB) [file 248_2022_2151_MOESM1_ESM.jpg]
